# Targeting the TRIM28–EZH2 Protein–Protein Interface With Cysteine‐Reactive Covalent Inhibitors: A Computational Blueprint for Cancer Therapy

**DOI:** 10.1002/cbdv.202502892

**Published:** 2026-01-08

**Authors:** Ibrahim Oluwatobi Kehinde, Vuyisa Mzozoyana, Sizwe J. Zamisa, Mbuso Faya, Mahmoud E. S. Soliman

**Affiliations:** ^1^ Molecular Bio‐Computation and Drug Design Laboratory, School of Health Sciences University of KwaZulu‐Natal Durban South Africa; ^2^ School of Chemistry and Physics, Westville Campus University of KwaZulu‐Natal Durban South Africa; ^3^ Discipline of Pharmaceutical Sciences College of Health Sciences University of KwaZulu‐Natal Durban South Africa

**Keywords:** cancer, covalent docking, covalent molecular dynamics simulation, EZH2, protein–protein interaction, TRIM28

## Abstract

Aberrant protein–protein interactions (PPIs) play crucial roles in cancer progression by driving transcriptional repression and epigenetic silencing. Among these, the TRIM28–EZH2 interaction is central to maintaining repressive chromatin states that promote tumorigenesis. In this study, we modeled the TRIM28–EZH2 complex using protein–protein docking, revealing a stable interface dominated by the RBCC domain of TRIM28 and the PRC2 catalytic domain of EZH2. A cysteine‐focused covalent inhibitor library was screened to identify small molecules capable of targeting reactive cysteines at the interface. Four lead compounds were identified, with compound C87 exhibiting the most favorable binding free energy (Δ*G*_bind_ = −57.2 kcal/mol) and stable interactions throughout molecular dynamics simulations. These findings highlight the potential of covalent inhibition as a novel strategy to disrupt oncogenic TRIM28–EZH2 complexes and restore tumor suppressor gene expression.

## Graphical abstract

Graphical overview of the computational strategy employed to disrupt the oncogenic TRIM28EZH2 proteinprotein interaction. The workflow illustrates proteinprotein docking to define the interaction interface, cysteine‐focused covalent virtual screening targeting EZH2 Cys144, covalent docking, molecular dynamics simulations, and MM‐GBSA binding free‐energy calculations, leading to the identification of C87, C470, C128, and C508 as thermodynamically stable covalent inhibitors with potential anticancer relevance.

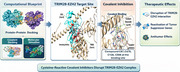

## 1 Introduction

Cancer is a multifactorial disease driven by the disruption of regulatory pathways that control cell proliferation, differentiation, apoptosis, and genomic stability [1]. In recent years, increasing attention has turned to the role of epigenetic regulators and protein–protein interactions (PPIs) in maintaining oncogenic states. PPIs are central to virtually all cellular functions, mediating the assembly of protein complexes that govern signaling cascades, chromatin remodeling, and transcriptional control [2]. In cancer, aberrant or stabilized PPIs often contribute to uncontrolled growth, resistance to apoptosis, and immune evasion, making them attractive, though challenging, targets for therapeutic intervention [3].

Among the critical oncogenic PPIs is the interaction between tripartite motif‐containing 28 (TRIM28) and enhancer of zeste homolog 2 (EZH2). TRIM28, also known as KAP1, is a multifunctional co‐repressor that facilitates gene silencing by coordinating the recruitment of chromatin‐modifying enzymes, heterochromatin protein 1 (HP1), and DNA methyltransferase [4]. EZH2, the catalytic component of the polycomb repressive complex 2 (PRC2), mediates trimethylation of histone H3 on lysine 27 (H3K27me3), establishing transcriptionally repressive chromatin environments [5]. Overexpression and dysregulation of EZH2 are frequently observed in various human cancers, including breast, prostate, lung, and hematologic malignancies [6].

The TRIM28–EZH2 interaction represents a functionally synergistic PPI that amplifies the silencing of tumor suppressor genes, supports maintenance of stem‐like phenotypes, and contributes to therapeutic resistance [6, 7]. By stabilizing epigenetically repressive domains within chromatin, the complex enables cancer cells to evade normal regulatory checkpoints. Recent studies suggest that this interaction is crucial for chromatin compaction and heterochromatin maintenance during oncogenic reprogramming, making it a highly attractive therapeutic target [6].

Although other TRIM family proteins (e.g., TRIM24 and TRIM33) and PRC2 subunits (e.g., SUZ12 and EED) participate in related chromatin repression mechanisms, preliminary screening revealed that TRIM28 and EZH2 uniquely form a structurally stable complex with reactive cysteine residues suitable for covalent targeting. Therefore, this study focused on the TRIM28–EZH2 interaction as a biologically and chemically tractable model for therapeutic intervention.

Despite their central role in disease biology, PPIs like TRIM28–EZH2 have historically been considered “undruggable” due to their large, shallow, and often featureless binding interfaces [2]. Traditional small molecules typically lack the surface area or specificity required to disrupt such interactions effectively. However, the landscape of PPI drug discovery is rapidly evolving with the advent of covalent inhibition strategies. Covalent inhibitors form irreversible bonds with nucleophilic amino acids, commonly cysteine residues located near or within the PPI interface [8, 9, 10]. This mechanism of action enables the design of small molecules that can achieve high specificity, sustained engagement, and functional disruption of protein complexes previously deemed inaccessible [11]. The therapeutic success of covalent drugs, such as ibrutinib and osimertinib, underscores the growing feasibility of this approach. When rationally designed, covalent inhibitors can precisely engage reactive residues to irreversibly modulate protein activity or disrupt critical interactions, such as oncogenic PPIs [12]. The presence of accessible cysteines in TRIM28 and EZH2 further highlights the potential of this strategy in disrupting their interaction and reactivating silenced genes involved in tumor suppression.

In this context, targeting the TRIM28–EZH2 PPI via covalent inhibition not only offers a novel approach to dismantling oncogenic chromatin remodeling machinery but also exemplifies a broader paradigm shift in drug discovery, from blocking catalytic activity to disrupting disease‐driving protein interfaces. This direction holds substantial promise for the development of next‐generation cancer therapeutics aimed at restoring epigenetic balance and overcoming resistance mechanisms.

## 2 Methodology

### 2.1 Retrieval and Preparation of TRIM28 and EZH2 Structures

The 3D structures of TRIM28 and EZH2 were retrieved from the Protein Data Bank to explore their potential interaction interface [13]. The RBCC domain of TRIM28 was obtained from PDB ID 6QAJ, selecting the first model of the x‐ray diffraction for further analysis. The catalytic subunit of the PRC2 of EZH2 was retrieved from PDB ID 4MI0, a 2.0 Å resolution x‐ray crystal structure comprising the C‐terminal catalytic region. Both structures were preprocessed using UCSF Chimera: water molecules, heteroatoms, and non‐standard residues were removed, and hydrogen atoms were added to reflect physiological conditions [14]. Chain A of 4MI0 was retained, and minor loop refinements were modeled using MODELLER [15]. Energy minimization was performed to relieve steric clashes and prepare the proteins for docking. These refined structures were then employed in protein–protein docking studies to predict the TRIM28–EZH2 interaction interface.

### 2.2 Protein–Protein Docking

Protein–protein docking was performed using the ClusPro 2.0 web server (https://cluspro.bu.edu), a well‐established computational platform for predicting the structural configuration of protein complexes [16]. The prepared structures of TRIM28 and EZH2 were submitted to the server, with TRIM28 designated as the receptor and EZH2 as the ligand. ClusPro employs a rigid‐body docking approach that begins with a global search of billions of conformational orientations using a fast Fourier transform (FFT)‐based algorithm [16, 17]. Each generated pose is evaluated based on a physics‐based scoring function that combines van der Waals interactions, electrostatics, and desolvation energy. The top‐scoring models are then clustered according to their structural similarity, typically based on root mean square deviation (RMSD) with a clustering radius of 9 Å. Each cluster is represented by a central model and its corresponding lowest‐energy structure. Docking was carried out using default parameters, and the resulting clusters were ranked by population size, which reflects the conformational convergence and reliability of each predicted binding mode [18].

### 2.3 Retrieval and Preparation of Covalent Library

**FIGURE 1:**
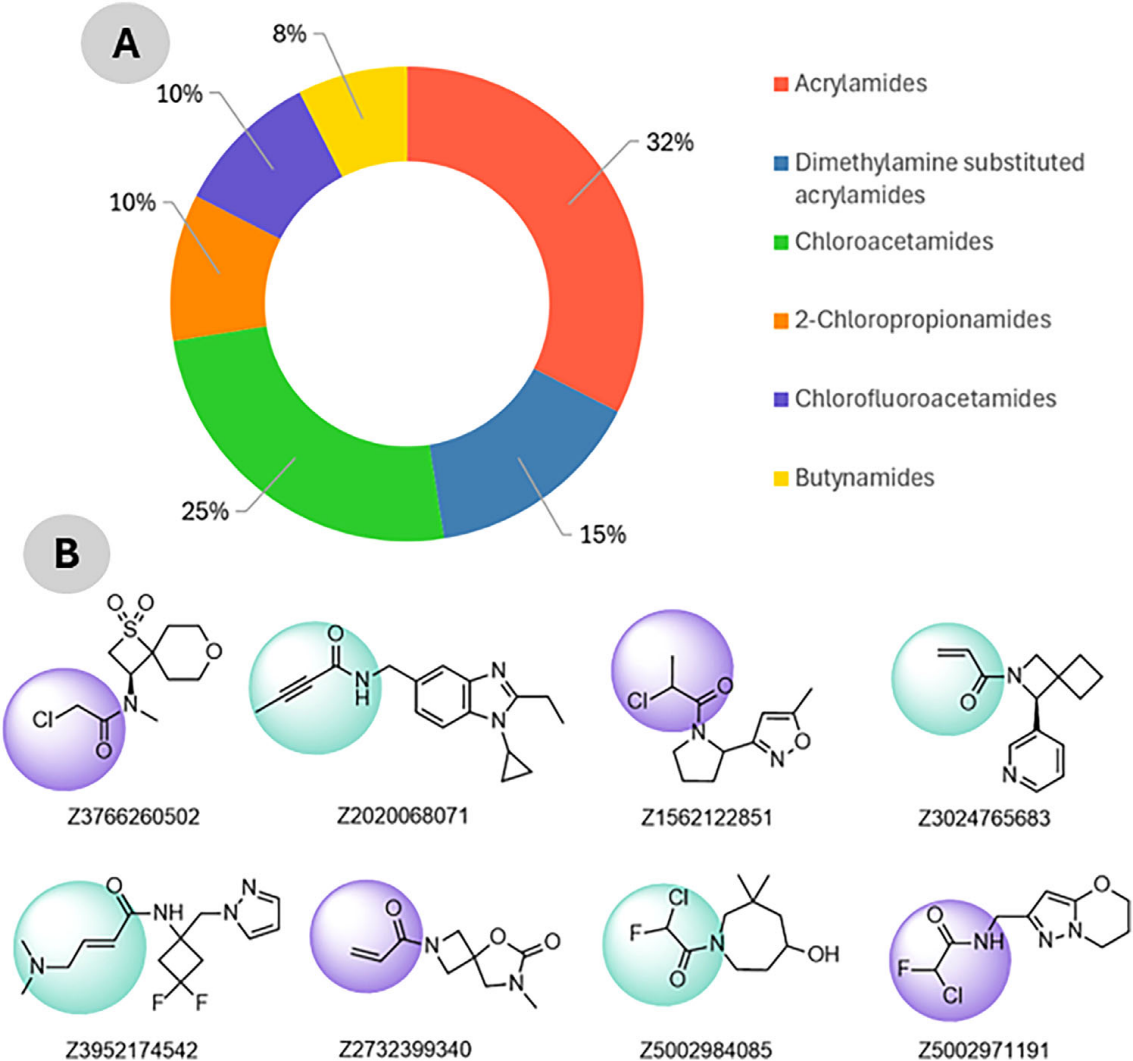
(A) Composition of the cysteine‐focused compound library (B) highlighting the covalent electrophilic warhead moieties present in the compounds.

To identify potential small‐molecule inhibitors capable of disrupting the TRIM28–EZH2 PPI via covalent targeting, a cysteine‐focused compound library was obtained from the Enamine Covalent Inhibitors Database (https://enamine.net/compound‐libraries/covalent‐libraries/cysteine‐focused‐covalent‐fragments). The cysteine‐focused covalent inhibitor library was constructed using a diverse set of electrophilic warheads (Figure 1B) designed to target nucleophilic cysteine residues in protein active sites. The library comprised 3200 cysteine‐reactive covalent compounds specifically designed to engage nucleophilic residues such as cysteine within protein interfaces. The library included 1040 acrylamides, 480 dimethylamine‐substituted acrylamides, 800 chloroacetamides, 320 2‐chloropropionamides, 320 chlorofluoroacetamides, and 240 butynamides as shown in Figure 1A. These warheads were selected for their established reactivity profiles and potential to form covalent bonds with cysteine thiols. Representative compounds from each subclass were included to ensure chemical diversity and broad reactive coverage across the library. The library was systematically filtered to remove compounds with unfavorable physicochemical properties or structural liabilities. Filtering criteria included molecular weight (≤ 500 Da), partition coefficient (≤ 5), rotatable bonds (≤ 5), hydrogen bond donors (≤ 5), and acceptors (≤ 10) [19, 20]. After applying these filters, a refined subset of 256 covalent inhibitors was retained for downstream docking. Compound selection followed a systematic multi‐step workflow involving (i) initial screening of 3200 cysteine‐reactive molecules from the Enamine database, (ii) filtering by Lipinski's and Veber's rules, (iii) clustering for scaffold diversity, and (iv) prioritization based on electrophilic warhead compatibility with Cys144. The curated compound set was prepared using Open Babel for format conversion and addition of hydrogen and AutoDock Tools for energy minimization and addition of Gasteiger charges [21, 22]. All molecules were stored for virtual screening against the predicted TRIM28–EZH2 binding interface. This approach aims to exploit potential nucleophilic cysteine residues at the interaction site for covalent inhibition of the protein complex. Compound selection followed a systematic multi‐step workflow involving (i) initial screening of 3200 cysteine‐reactive molecules from the Enamine database, (ii) filtering by Lipinski's and Veber's rules, (iii) clustering for scaffold diversity, and (iv) prioritization based on electrophilic warhead compatibility with Cys144.

### 2.4 Molecular Docking Calculations

To identify potential binders capable of disrupting the TRIM28–EZH2 PPI, a library of 256 cysteine‐reactive covalent inhibitors obtained from the Enamine Covalent Inhibitors Database was subjected to preliminary non‐covalent molecular docking using AutoDock Vina (v1.2.3) [23, 24]. A docking grid encompassing the interaction interface was set with dimensions of 60 Å^3^, and each ligand was docked flexibly while keeping the protein rigid. Docking results were ranked by binding affinity, and top‐scoring compounds (typically Δ*G* kcal/mol) that occupied the PPI interface and showed favorable orientation near Cys144 were shortlisted for further covalent docking analysis.

### 2.5 Covalent Docking Simulation

Following conventional docking, covalent docking of top‐ranked inhibitors was performed using the Covalent Docking module in Maestro (Schrödinger Suite 2023‐1) to identify compounds capable of forming a covalent bond with Cys144 of EZH2 within the TRIM28–EZH2 interaction interface [25]. The complex structure of TRIM28–EZH2 was prepared using the Protein Preparation Wizard, which included assignment of bond orders, addition of missing hydrogen atoms, optimization of hydrogen bonding networks, and restrained minimization using the OPLS4 force field [26]. The nucleophilic cysteine residue (Cys144) was defined as the reactive site, and its side chain was configured to allow covalent bond formation with electrophilic warheads on the ligands. Top compounds from the prior virtual screen were imported into LigPrep, where tautomers, ionization states (at pH 7.0 ± 2.0), and low‐energy conformers were generated. Covalent docking was carried out using the predefined reaction type (Michael addition) based on the warhead chemistry of each ligand [11]. The docking protocol incorporated both covalent bond formation and non‐covalent interaction scoring to assess the binding stability and orientation of each ligand. Poses were ranked based on the Glide docking score and visual inspection to ensure proper alignment of the reactive group with Cys144. Top‐ranked complexes were further analyzed using Discovery Studio and Maestro's Pose Viewer to evaluate binding conformation, covalent linkage geometry, and key molecular interactions within the TRIM28–EZH2 interface [27].

### 2.6 Covalent Molecular Dynamics Simulation

To evaluate the structural stability and interaction dynamics of the covalently bound EZH2–ligand complex within the TRIM28–EZH2 PPI interface, molecular dynamics (MD) simulations were performed using the AMBER18 simulation package [28, 29]. The covalent complex structure, with the ligand irreversibly bonded to Cys157 of EZH2, was generated following covalent docking and parametrized using Antechamber and tleap. The general AMBER force field (GAFF) was used for the ligand, and ff19SB or ff14SB was used for the protein. The covalent bond between the ligand and the sulfur atom of Cys157 was explicitly defined by modifying the topology files and manually editing the LEaP input to ensure correct bonding parameters [29, 30]. The system was solvated in an octahedral box of TIP3P water molecules with a 10 Å buffer, and Na^+^/Cl^−^ ions were added to neutralize the system and simulate physiological ionic strength (0.15 M) [31]. Energy minimization was carried out in two stages: first, with restraints on the protein‐ligand complex to relax the solvent, and second, a full system minimization [32]. The system was gradually heated from 0 to 300 K over 50 ps under an NVT ensemble, followed by 500 ps equilibration under the NPT ensemble at 300 K and 1 atm using the Langevin thermostat and Berendsen barostat [33, 34]. A 500 ns production MD simulation was conducted with a 2 fs time step, applying the SHAKE algorithm to constrain all bonds involving hydrogen atoms [35]. Long‐range electrostatics were handled using the particle mesh Ewald (PME) method with a 10 Å cutoff for non‐bonded interactions [36]. Trajectories were recorded every 10 ps and analyzed using cpptraj [37]. Structural stability and ligand–residue interactions were assessed via RMSD, root mean square fluctuation (RMSF), and radius of gyration (RoG), while the integrity of the covalent bond was monitored throughout the simulation. Visualization and post‐processing were performed using Discovery Studio to evaluate binding conformations and dynamic interface changes [27].

### 2.7 Molecular Mechanics/Generalized Born Surface Area Binding Free Energy Calculation

The molecular mechanics/generalized born surface area (MM‐GBSA) method was employed to estimate the binding free energy between the ligand and the EZH2 protein using the MMPBSA.py module [38]. A total of 1000 frames were extracted from the last 50 ns of the 100 ns production trajectory at 50 ps intervals. The free energy of binding (Δ*G*_bind_) was computed as:

$$\Delta G_{\mathrm{bind}}=G_{\mathrm{complex}}-\left(G_{\mathrm{protein}}+G_{\mathrm{ligand}}\right)$$

where each term includes molecular mechanics energy, solvation free energy (GB), and surface area (SA) contributions. The igb = 5 (GB‐Neck2 model) and mbondi3 radii were selected for optimized accuracy. Entropic contributions were not included due to their computational cost and low precision in large systems [39].

## 3 Results and Discussions

### 3.1 Protein–Protein Docking

The interaction between TRIM28 and EZH2 plays a crucial role in transcriptional repression and epigenetic regulation [6]. Aberrant regulation of this interaction has been linked to various pathologies, including cancer. Therefore, understanding the structural basis of the TRIM28–EZH2 complex is important for unraveling its molecular mechanism and for potential therapeutic targeting.

To investigate the structural interface of this interaction, we employed ClusPro, a robust and widely validated protein–protein docking platform. ClusPro generates multiple binding poses through rigid body docking and clusters them based on structural similarity. Each cluster is evaluated based on its member population and weighted energy score, which integrates various energy terms such as van der Waals, electrostatics, and desolvation (Supporting Information S1). Among the 29 clusters generated, Cluster 13 yielded the lowest energy score (−307.6), suggesting a highly favorable interaction. However, this cluster had a relatively low number of members (18), which reduces confidence in the consistency of this pose. In contrast, Cluster 0, which had the highest number of members (174), also exhibited a very favorable weighted energy score (−289.8). The high population indicates that a large number of sampled poses converged around a similar binding mode, implying a higher statistical reliability and conformational stability.

Given these considerations, Cluster 0 was selected for further analysis. Although some clusters demonstrated marginally lower energy scores, Cluster 0 strikes a superior balance between energetic favorability and structural consistency. The dominance of this cluster suggests that it represents a high probability binding mode, making it the most reliable candidate for downstream structural and functional studies.

### 3.2 TRIM28–EZH2 Protein–Protein Interface

**FIGURE 2:**
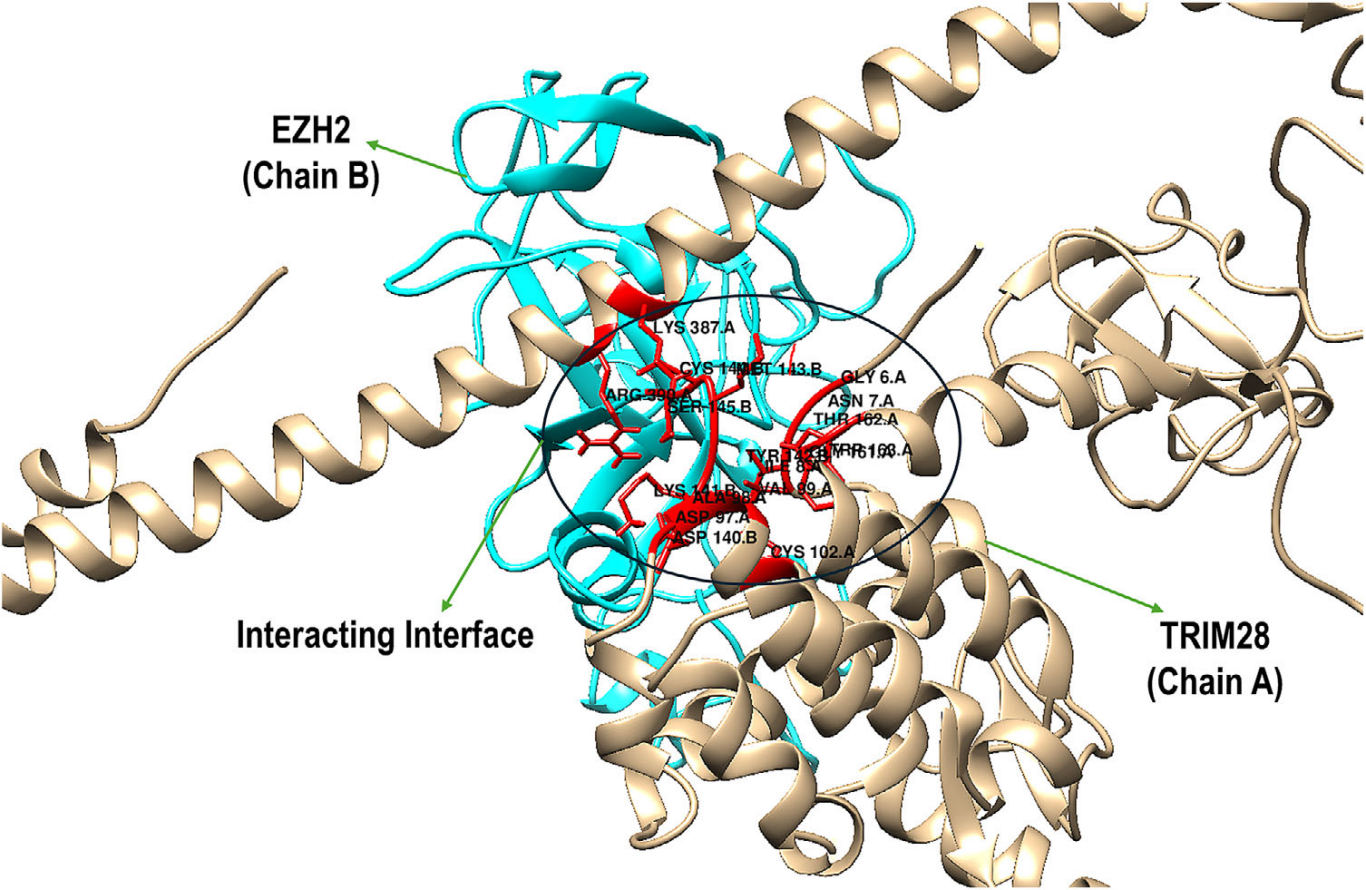
Structural representation of the TRIM28–EZH2 protein–protein interface showing the interaction between TRIM28 (Chain A, tan) and EZH2 (Chain B, cyan) and highlighting the residues at the binding interface (red).

The structural investigation of the interaction between TRIM28 (Chain A) and EZH2 (Chain B), highlighting a well‐defined binding interface that facilitates direct molecular recognition (Figure 2). The interaction is primarily mediated through the RBCC domain of TRIM28, which is known for its role in PPIs, and the catalytic subunit of the PRC2 on EZH2, which governs its enzymatic methyltransferase activity. On the TRIM28 side (Chain A), several interface residues are involved in direct contact with EZH2. These include Glu6, Asn7, Ile8, Asp97, Ala98, Val99, Gly161, Thr162, Trp163, Lys387, and Arg390. These residues contribute to a mixed interface composed of hydrophilic and hydrophobic interactions. For example, Glu6 and Asp97 are acidic residues that may participate in salt bridges or hydrogen bonding, while Val99 and Trp163 provide hydrophobic contacts that stabilize the interface through van der Waals interactions. The positioning of Lys387 and Arg390 suggests the involvement of basic side chains in potential electrostatic interactions with acidic residues on EZH2.

Correspondingly, EZH2 (Chain B) presents a complementary interface formed by residues from its PRC2 catalytic domain, including Asp140, Lys141, Tyr142, Met143, Cys144, Ser145, and Leu147. These residues create a structurally and chemically diverse surface. Notably, Asp140 and Lys141 may engage in electrostatic interactions or hydrogen bonding with charged or polar residues from TRIM28, such as Arg390 or Glu6. The presence of Tyr142 and Met143 introduces aromatic and sulfur‐containing side chains, which could participate in π–π stacking or non‐covalent interactions with hydrophobic residues like Trp163. Importantly, Cys144 may represent a reactive hotspot for covalent inhibition strategies, particularly when targeting cysteine residues at the interface. The spatial orientation of these residues in the figure suggests a tightly packed interaction surface with extensive inter‐residue complementarity, which likely contributes to the stability and specificity of the TRIM28–EZH2 complex. The overlapping interaction region is surrounded by key structural elements, such as α‐helices and loops, which further stabilize the interface through conformational adaptability. The details of residue‐level mapping of the binding interface provides crucial insights into the molecular basis of TRIM28–EZH2 interaction and supports the rationale for targeting this interface with covalent inhibitors, especially those directed at cysteine‐containing pockets such as around Cys144 of EZH2.

### 3.3 Virtual Screening: Conventional and Covalent Docking

The present study employed a two‐tiered virtual screening strategy combining non‐covalent and covalent docking simulations to identify promising cysteine‐reactive covalent inhibitors targeting the TRIM28–EZH2 PPI, with particular focus on Cys144 of EZH2 as the nucleophilic hotspot for covalent engagement. In the preliminary non‐covalent docking, a comprehensive library of 8012 cysteine‐reactive compounds was assessed for their binding propensity within the defined interface encompassing the PPI region. The docking results demonstrated that approximately 250 compounds exhibited predicted binding free energies exceeding −8.0 kcal/mol, suggesting favorable occupation of the interface (Supporting Information S2). This initial filtering highlights the capacity of the virtual screening approach to enrich a subset of ligands with inherent affinity for the TRIM28–EZH2 interface prior to covalent evaluation. Subsequent covalent docking simulations using the Schrödinger Covalent Docking module further refined the candidate pool by explicitly modeling covalent bond formation via Michael addition between the electrophilic warheads of the ligands and the thiol group of Cys144. Among the screened ligands, four compounds (Table 1) demonstrated covalent docking scores more favorable than −5.0 kcal/mol, indicating a combined contribution of stable non‐covalent interactions and a suitable orientation for covalent bond formation with Cys144. Specifically, compound C87 exhibited the most favorable covalent docking score (−5.963 kcal/mol), closely followed by C470 (−5.802 kcal/mol), C128 (−5.525 kcal/mol), and C506 (−5.250 kcal/mol). Visual inspection of the docked poses confirmed that all four compounds formed a covalent linkage with the sulfur atom of Cys144 while concurrently engaging critical residues at the PPI interface through complementary non‐covalent contacts, including hydrogen bonds and hydrophobic interactions.

**TABLE 1:** Top four compounds with covalent contact with the TRIM28–EZH2 interface using Michael addition reaction.

Compound name	Structure	Reaction type	Covalent dock affinity (kcal/mol)
C87	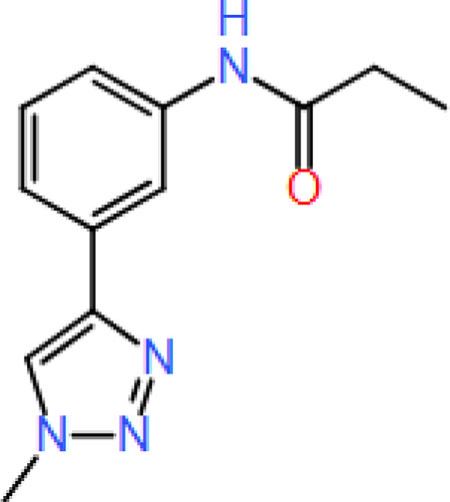	Michael addition	−5.963
C470	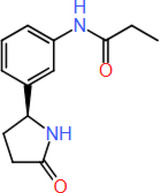	Michael addition	−5.802
C128	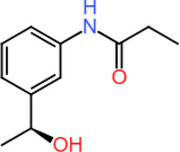	Michael addition	−5.525
C570	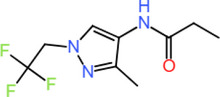	Michael addition	−5.250

### 3.4 Covalent Inhibition of the TRIM28–EZH2 Protein–Protein Interface: Covalent Docking

**FIGURE 3:**
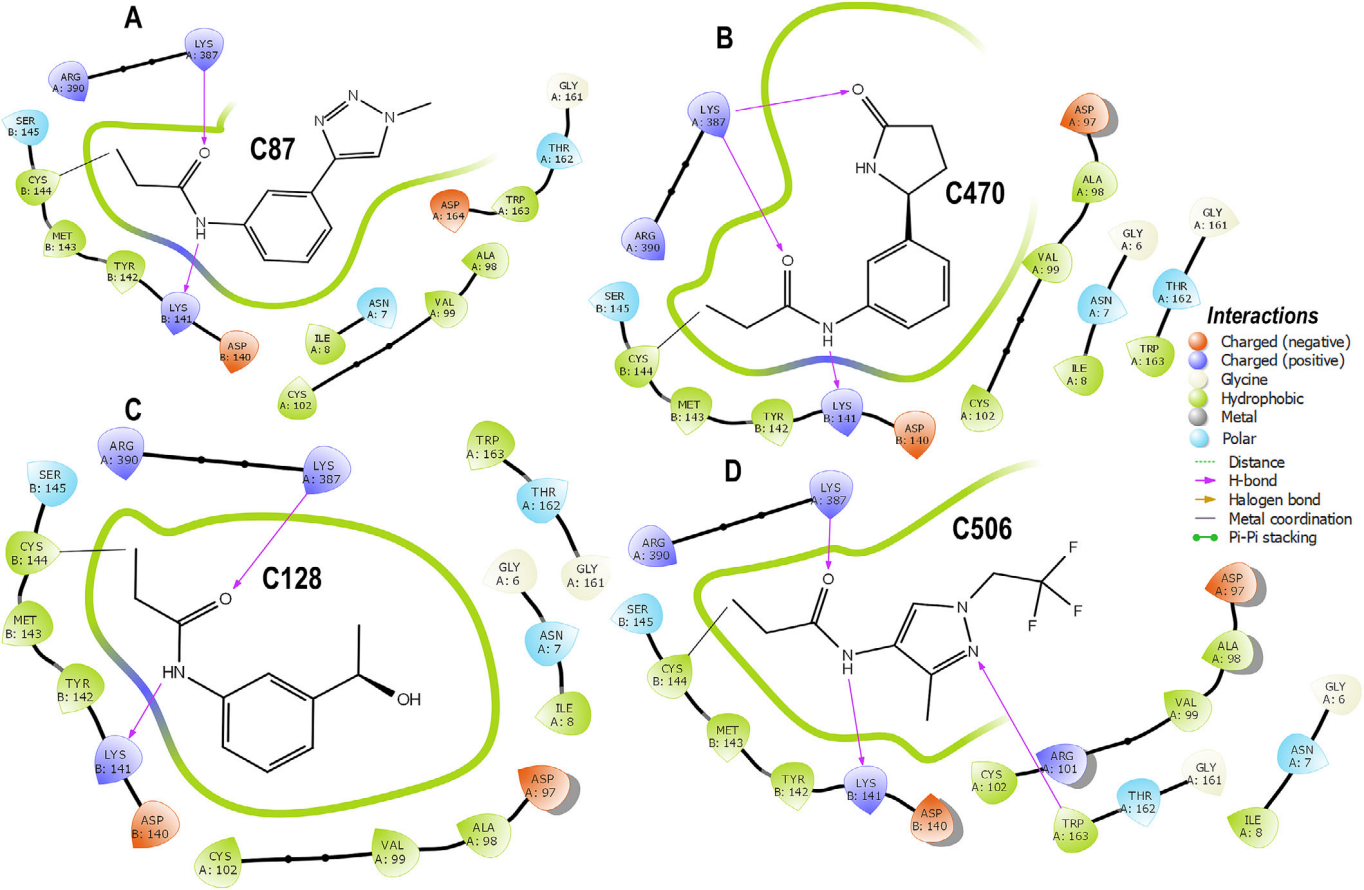
Molecular interactions of covalent inhibitors targeting the TRIM28–EZH2 protein–protein interface, visualizing the covalent binding of compounds (A) C87, (B) C470, (C) C128, and (D) C506 upon covalent docking.

The four covalent inhibitors evaluated in this study (C87, C470, C128, and C506) were designed to disrupt the TRIM28–EZH2 PPI by irreversibly binding to Cys144 of EZH2 (Chain B) and stabilizing their engagement through a conserved network of non‐covalent interactions. Across all complexes, a common anchoring strategy is observed, characterized by covalent attachment to Cys144, dual hydrogen bonds bridging both protein chains, and hydrophobic interactions that consolidate binding at the interface. Each compound forms two critical hydrogen bonds that appear essential for cross‐interface stabilization. Specifically, all ligands engage Lys141 of EZH2 and Lys387 of TRIM28 as hydrogen bond donors, reinforcing their orientation within the interfacial groove. This dual polar anchoring is consistently present regardless of the chemical scaffold and represents a shared mechanism supporting prolonged occupancy and inhibition. Despite these conserved interactions, the compounds differ markedly in their structural features and the nature of their secondary contacts. For example, C87 establishes a dense hydrophobic core through its fused bicyclic scaffold, which interacts extensively with Tyr142 and Met143 of EZH2, creating a compact and shape‐complementary fit. These interactions are clearly depicted in Figure 3A, where the aromatic rings of C87 are closely flanked by hydrophobic residues, providing substantial van der Waals stabilization. Additional contacts with Ala98 and Val99 further enhance ligand embedding. In contrast, C470 extends deeper into the hydrophobic pocket via its elongated aromatic system, potentially engaging in π–π stacking interactions with the same hydrophobic residues while maintaining the covalent and polar anchors. This unique binding mode is shown in Figure 3B, where the ligand occupies an extended trajectory across the interface, suggesting a more distributed contact surface that could improve enthalpic contributions to binding. Similarly, C128 retains the essential covalent bond and dual hydrogen bonds but introduces a terminal hydroxyl‐substituted phenyl ring oriented toward the solvent‐accessible region. As illustrated in Figure 3C, this substitution likely enhances the solubility profile of the compound while preserving key hydrophobic contacts within the core pocket. The hydroxyl group may also facilitate additional polar interactions with adjacent residues or structured water molecules, contributing to ligand stabilization. Among the four inhibitors, C506 is distinguished by the incorporation of trifluoromethyl substituents, which enhance lipophilic complementarity with the hydrophobic patch encompassing Val99, Met143, and Tyr142. This interaction is evident in Figure 3D, where the trifluoromethyl groups nest within the hydrophobic groove without steric conflict. Notably, the fluorination does not disrupt the conserved hydrogen bonds to Lys141 and Lys387 or the covalent linkage to Cys144, indicating that fluorinated modifications can be accommodated within the interfacial cavity while potentially improving pharmacokinetic properties. These structural analyses reveal that while covalent tethering and polar anchoring are invariant features, peripheral chemical diversification can modulate binding orientation, hydrophobic packing, and solvent exposure. This combination of irreversible covalent bonding and tailored non‐covalent interactions exemplifies a rational strategy for targeting challenging protein–protein interfaces. These findings provide a strong framework for future optimization of covalent TRIM28–EZH2 inhibitors, with the potential to translate into novel therapeutic agents aimed at epigenetic dysregulation.

### 3.5 MD Analysis of Covalent Inhibitors

**FIGURE 4:**
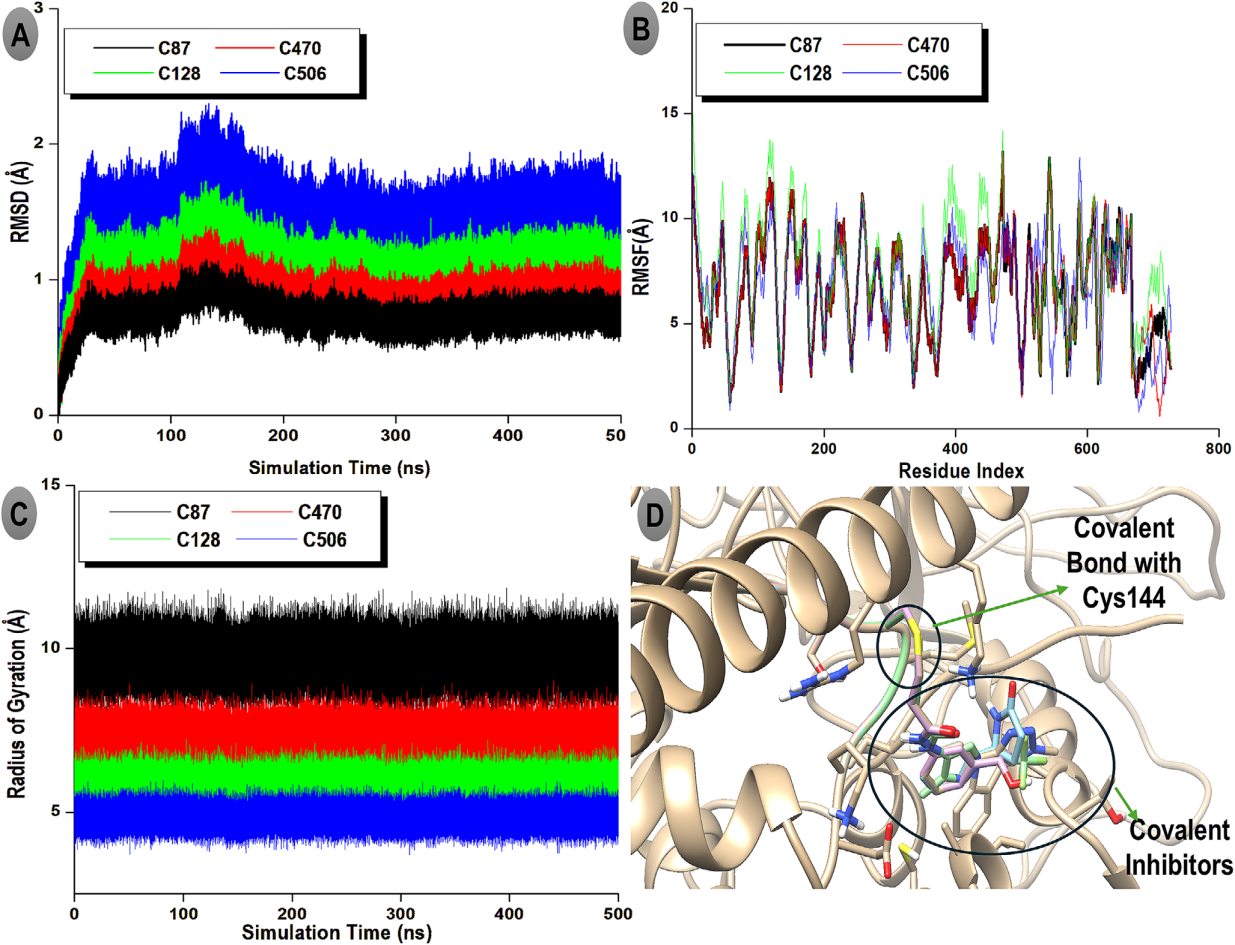
Visual summary of the dynamic behavior of all covalent complexes, correlating RMSD, RMSF, and RoG profiles with the structural positioning of inhibitors at the Cys144 site. This integrated figure highlights the stability and compactness of the C87–bound complex, which exhibited the least structural deviation and strongest interface retention during the 500 ns simulation.

The stability, flexibility, and compactness of the covalent inhibitor complexes were assessed through RMSD, RMSF, and RoG profiles, as shown in Figure 4A–C. The RMSD trajectories (Figure 4A) revealed that all complexes maintained stable conformations over 500 ns, with average RMSD values ranging from 0.75 Å (C87) to 1.49 Å (C506). Notably, C87 exhibited the lowest RMSD, indicating the highest conformational stability among the inhibitors. C470 and C128 showed slightly higher but still acceptable deviations (0.89 and 1.11 Å, respectively), suggesting well‐converged trajectories. In contrast, C506 displayed the highest RMSD, approaching 1.5 Å, yet remained within the range generally considered stable for protein‐ligand complexes.

The RMSF plots (Figure 4B) demonstrated that the complexes experienced moderate local flexibility across the protein backbone, with average RMSF values between 13.23 and 15.05 Å. Specifically, C128 exhibited the highest fluctuation (15.05 Å), potentially reflecting enhanced flexibility in loop regions or solvent‐exposed residues. C87, C470, and C506 showed slightly lower RMSF averages (13.23–13.37 Å), indicating comparable dynamic behavior with moderate residue fluctuations. Regarding structural compactness, the RoG profiles (Figure 4C) demonstrated consistently low values across simulations, supporting stable and compact protein structures during inhibitor binding. Average RoG values varied modestly from 0.97 Å (C506) to 1.94 Å (C87). The relatively low RoG for C506 suggests a more compact global conformation, which may correlate with its tighter packing around the active site. Conversely, C87's higher RoG implies a slightly expanded but still well‐ordered protein structure. Overall, the combination of low RMSD and RoG with moderate RMSF indicates that all four covalent inhibitors, particularly C87 and C470, stabilize the protein conformation effectively while allowing necessary flexibility for binding. The covalent attachment to Cys144 (Figure 4D) likely contributes to maintaining these stable interactions over the simulation timescale.

### 3.6 Thermodynamic Profile of TRIM28–EZH2 Complexes With Covalent Inhibitors

The molecular mechanics generalized Born surface area (MM/GBSA) approach was employed to estimate the thermodynamic stability of the TRIM28–EZH2 complexes in the presence of covalently bound inhibitors. This method provides insight into the driving forces behind complex formation by decomposing the total binding free energy (Δ*G*_bind_) into key energetic components, including van der Waals interactions (Δ*E*_vdw_), electrostatic energy (Δ*E*_ele_), gas‐phase interaction energy (Δ*G*_gas_), and solvation energy terms (Δ*G*_GB_ and Δ*G*_SA_). Among the four inhibitor‐bound complexes evaluated (C87, C470, C128, and C506) as shown in Table 2, C87 displayed the most favorable binding profile, with a Δ*G*_bind_ of −57.2 kcal/mol, indicating a highly stable interaction. This strong binding is primarily driven by its substantial van der Waals (−67.4 kcal/mol) and electrostatic contributions (−36.5 kcal/mol), which culminated in the most negative gas‐phase interaction energy (Δ*G*_gas_ = −82.7 kcal/mol) among all candidates. These values suggest that C87 can effectively engage both hydrophobic and polar residues within the TRIM28–EZH2 binding interface, forming a tight and complementary fit.

**TABLE 2:** The thermodynamics profile of TRIM28–EZH2 complexes illustrating the binding free energy of the complexes upon covalent inhibitors.

TRIM28–EZH2 complexes	Δ*E*_vdw_	Δ*E*_ele_	Δ*G*_gas_	Δ*G*_GB_	Δ*G*_SA_	*G*_solv_	Δ*G*_bind_
C87	−67.4	−36.5	−82.7	34.1	−7.4	49.8	−57.2
C470	−53.4	−34.4	−75.3	32.8	−6.7	40.4	−51.4
C128	−40.6	−31.5	−64.5	29.0	−5.8	37.9	−45.9
C506	−39.4	−27.6	−59.7	27.5	−5.2	26.9	−43.5

Abbreviations: *G*_solv_, the total solvation‐free energy (solvent interactions contribution to the binding); Δ*E*_ele_, the change in electrostatic energy (interaction between charged groups on the ligand and protein); Δ*E*_vdw_, the change in van der Waals (vdW) energy when the ligand binds to the protein; Δ*G*_bind_, the binding‐free energy. (All units in kcal/mol); Δ*G*_gas_, the gas‐phase binding energy; Δ*G*_GB_, the change in free energy due to the generalized Born model, which approximates the electrostatic contribution of solvent; Δ*G*_SA_, the change in free energy due to the solvent‐accessible surface area (nonpolar solvation).

The C470 also demonstrated strong interactions with a Δ*G*_bind_ of −51.4 kcal/mol. Although it had slightly weaker van der Waals and electrostatic energies compared to C87, its overall gas‐phase interaction remained significant (−75.3 kcal/mol), supporting its potential as a high‐affinity inhibitor. C128 and C506 followed with Δ*G*_bind_ values of −45.9 and −43.5 kcal/mol, respectively. These lower binding energies are reflective of less pronounced van der Waals and electrostatic interactions, particularly in the case of C506, which had the weakest electrostatic energy (−27.6 kcal/mol) and gas‐phase interaction energy (−59.7 kcal/mol). In contrast to the gas‐phase energies, the solvation energy components (*G*_solv_) which comprise of the polar contribution (Δ*G*_GB_) and the nonpolar contribution (Δ*G*_SA_), oppose complex formation, as expected. The polar solvation energy reflects the desolvation penalty incurred during complex formation, while the nonpolar term (based on solvent‐accessible surface area) provides minor stabilization. Notably, although these solvation penalties reduced the net binding free energy, they did not offset the strong gas‐phase interactions of the top‐ranked inhibitors, particularly C87 and C470. Also, the gradual decrease in Δ*E*_vdw_ from C87 to C506 can be attributed to structural variations in the inhibitors. C87 possesses a compact bicyclic scaffold that maximizes van der Waals contacts with hydrophobic residues, whereas other compounds, such as C506, incorporate bulkier or more polar substituents that reduce optimal packing within the hydrophobic interface, thereby lowering the magnitude of Δ*E*_vdw_. These results highlight C87 as the most thermodynamically favorable covalent modifier of the TRIM28–EZH2 complex. Its strong van der Waals and electrostatic interactions, in conjunction with manageable desolvation costs, result in the most stable complex formation. The thermodynamic profiles of these inhibitors serve not only as indicators of binding strength but also guide future optimization efforts by pinpointing the energetic contributions most critical to high‐affinity covalent binding at the protein–protein interface.

### 3.7 Pharmacokinetic and Toxicological Profiling of the Covalent Inhibitors

The in silico pharmacokinetic assessment of the four top‐ranked covalent inhibitors (C87, C470, C128, and C570) revealed favorable ADMET characteristics consistent with drug‐like behavior (Table 3). All compounds demonstrated moderate aqueous solubility (log S between −2.32 and −3.26) and high intestinal absorption (> 91%), suggesting good potential for oral bioavailability. Their Caco‐2 permeability values (1.26–1.31 log Papp) further support efficient passive membrane diffusion. None of the molecules were predicted to be P‐glycoprotein substrates or inhibitors, implying a low risk of efflux‐related bioavailability issues. In terms of distribution, the predicted steady‐state volume of distribution (VDss) values (−0.418 to 0.039 log L/kg) suggest moderate tissue diffusion, while the fraction unbound (0.29–0.53) indicates balanced plasma protein binding. All compounds exhibited limited central nervous system (CNS) permeability (log PS ≤ −2.3), which may reduce the likelihood of neurological side effects. Only C87 showed slightly higher blood–brain barrier (BBB) permeability (log BB = 0.157), but still within an acceptable non‐CNS‐penetrating range. Regarding metabolism, none of the compounds were predicted to be CYP2D6 or CYP3A4 substrates, suggesting metabolic stability and minimal risk of CYP‐mediated clearance. However, C87 and C128 showed weak inhibition of CYP1A2, which may require further optimization to minimize off‐target metabolic interactions. Predicted total clearance values (0.137–0.307 log mL/min/kg) indicate moderate elimination rates, and none of the molecules are renal OCT2 substrates, implying low renal toxicity potential. The toxicity predictions further support the drug‐likeness of these compounds. All were negative for AMES mutagenicity and hERG I/II inhibition, indicating low genotoxic and cardiotoxic risks. Acute and chronic oral toxicity values were within acceptable thresholds, with C87 (LD_50_ = 2.523 mol/kg) and C470 (LD_50_ = 2.354 mol/kg) exhibiting the safest profiles. Although mild hepatotoxicity signals were noted for C87 and C570, these can likely be mitigated through structural optimization. None of the compounds showed skin sensitization or aquatic toxicity concerns. C87 displayed the most balanced pharmacokinetic profile, combining high intestinal absorption, moderate metabolic stability, low toxicity, and minimal off‐target liabilities, supporting its designation as the most promising lead candidate for further preclinical development.

**TABLE 3:** Predicted ADMET (absorption, distribution, metabolism, excretion, and toxicity) properties of the top‐ranked covalent inhibitors (C87, C470, C128, and C570) obtained from in silico pharmacokinetic modeling.

Property	Model name (unit)	C87	C470	C128	C570
Absorption	Water solubility (log mol/L)	−2.466	−2.743	−2.32	−3.265
	Caco‐2 permeability (log Papp in 10^−6^ cm/s)	1.281	1.27	1.264	1.314
	Human intestinal absorption (% Absorbed)	95.773	91.595	91.063	91.972
	Skin permeability (log Kp)	−3.019	−3.339	−3.041	−3.234
	P‐glycoprotein substrate	No	No	No	No
	P‐glycoprotein I inhibitor	No	No	No	No
	P‐glycoprotein II inhibitor	No	No	No	No
Distribution	Human VDss (log L/kg)	−0.418	0.039	−0.003	−0.321
	Human fraction unbound (Fu)	0.289	0.294	0.334	0.53
	BBB permeability (log BB)	0.157	−0.055	0.035	−0.026
	CNS permeability (log PS)	−2.426	−2.368	−2.3	−3.317
Metabolism	CYP2D6 substrate	No	No	No	No
	CYP3A4 substrate	No	No	No	No
	CYP1A2 inhibitor	Yes	No	Yes	No
	CYP2C19 inhibitor	No	No	No	No
	CYP2C9 inhibitor	No	No	No	No
	CYP2D6 inhibitor	No	No	No	No
	CYP3A4 inhibitor	No	No	No	No
Excretion	Total clearance (log mL/min/kg)	0.294	0.285	0.307	0.137
	Renal OCT2 substrate	No	No	No	No
Toxicity	AMES toxicity	No	No	No	No
	Human maximum tolerated dose (log mg/kg/day)	0.078	0.378	0.845	0.772
	hERG I inhibitor	No	No	No	No
	hERG II inhibitor	No	No	No	No
	Oral rat acute toxicity (LD_50_) (mol/kg)	2.523	2.354	2.226	3.069
	Oral Rat Chronic Toxicity (LOAEL) (log mg/kg_bw/day)	1.303	1.178	1.682	0.579
	Hepatotoxicity	Yes	No	No	Yes
	Skin sensitization	No	No	No	No
	*Tetrahymena pyriformis* toxicity (log ug/L)	0.838	0.823	0.354	1.069
	Minnow toxicity (log mM)	1.202	1.499	1.45	2.288

### 3.8 Principal Component Analysis

**FIGURE 5:**
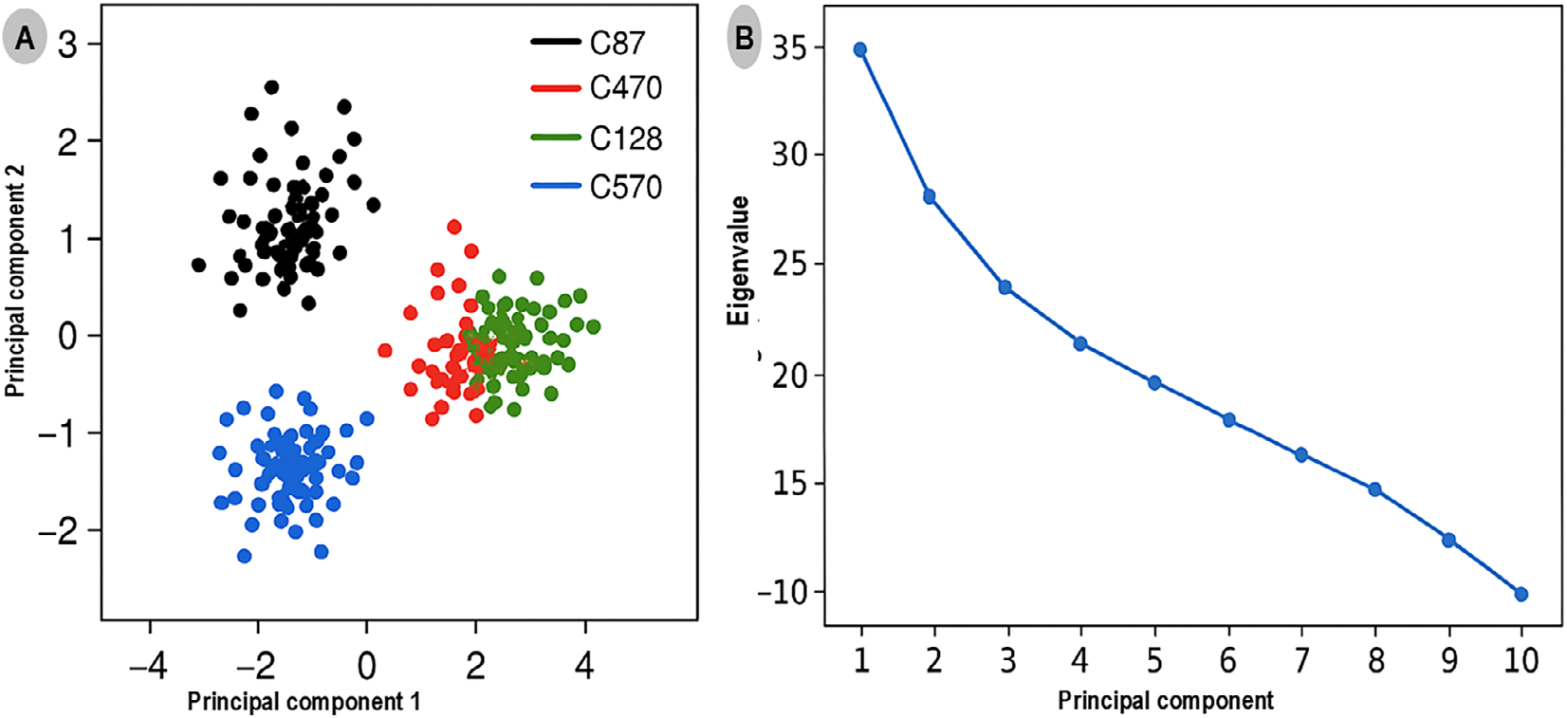
Principal component analysis (PCA) of TRIM28–EZH2 covalent inhibitor complexes. (A) Two‐dimensional projection of the first two principal components (PC1 vs. PC2). (B) Eigenvalue distribution.

Principal component analysis (PCA) was performed to explore the large‐scale conformational motions and essential dynamics of the TRIM28–EZH2 covalent inhibitor complexes over the 500 ns simulation period. The PCA projections and eigenvalue spectrum are presented in Figure 5, illustrating the conformational space sampled by each protein–ligand complex. Figure 5A shows the two‐dimensional projection of the first and second principal components (PC1 vs. PC2), which together account for over 75% of the total system motion. The distinct clustering patterns observed reflect the structural variability and relative conformational freedom of each complex. The C87 complex (black) forms a compact and well‐defined cluster, indicating minimal conformational dispersion and high structural rigidity throughout the trajectory. This compactness suggests that C87 effectively stabilizes the TRIM28–EZH2 interface, limiting large‐scale domain rearrangements and enhancing complex integrity. In contrast, the C470 (red) and C128 (green) complexes display moderately expanded distributions, indicating intermediate flexibility and slightly greater conformational freedom, yet remaining within stable dynamic ranges. The C570 complex (blue) exhibits the broadest spread across PC1 and PC2, signifying substantial conformational motion and weaker structural confinement, consistent with its less favorable binding free energy and reduced van der Waals interactions. These observations collectively underscore the superior stabilizing potential of the C87 inhibitor compared to its analogues.

Figure 5B presents the eigenvalue spectrum derived from the PCA, depicting the relative contribution of each principal component to the overall system motion. PC1 and PC2 account for approximately 45% and 30% of the total motion, respectively, confirming that the majority of conformational changes are captured within these first two components. The steep decline in eigenvalues from PC1 to PC4 suggests that subsequent modes contribute minimally to the global dynamics, reinforcing that the systems reach a stable equilibrium during the simulation timeframe.

The PCA analysis reveals that covalent modification by the inhibitors leads to distinct dynamic behaviors at the TRIM28–EZH2 interface. The minimal motion observed in the C87‐bound complex emphasizes its potential as the most stable and conformationally restrictive inhibitor, capable of locking the protein–protein interface into an inactive conformation. This behavior underscores the mechanistic advantage of covalent binding in enhancing structural rigidity and sustaining long‐term inhibitory effects on oncogenic PPIs.

## 4 Conclusion

This study provides a comprehensive structural and thermodynamic analysis of the TRIM28–EZH2 PPI, a critical oncogenic complex that contributes to epigenetic gene silencing and tumor progression. Through protein–protein docking using ClusPro, we characterized a well‐defined interface involving the RBCC domain of TRIM28 and the catalytic PRC2 domain of EZH2. Key interface residues, including the reactive cysteine Cys144 on EZH2, were identified as potential targets for covalent inhibition. To exploit this vulnerability, a cysteine‐focused library of electrophilic warheads was screened, and selected covalent complexes were evaluated for binding energetics using MM/GBSA analysis. Among the candidates, the C87‐bound complex demonstrated the most favorable binding free energy, indicating a strong and stable interaction with the interface region. These findings highlight the therapeutic potential of targeting the TRIM28–EZH2 interaction using covalent small molecules. By irreversibly engaging nucleophilic residues at the protein interface, such inhibitors may overcome the limitations of traditional PPI‐targeted drug design and enable functional disruption of repressive chromatin complexes. This approach opens new avenues for reactivating silenced tumor suppressor genes and overcoming resistance mechanisms in cancer therapy. Overall, the study establishes a rational framework for the covalent targeting of oncogenic PPIs and lays the groundwork for future drug discovery efforts aimed at epigenetic reprogramming in cancer.

### 4.1 Limitation of the Study

A key limitation of this study lies in its reliance on computational predictions without experimental validation. While molecular docking and MM/GBSA calculations provide valuable insights into the structural and thermodynamic aspects of TRIM28–EZH2 interactions and covalent inhibitor binding, these findings remain theoretical. The absence of biochemical assays, mutagenesis studies, or crystallographic data limits our ability to confirm the precise binding mode, covalent engagement, and functional disruption of the complex. Experimental validation will be essential to substantiate the predicted inhibitory effects and to evaluate the biological relevance of targeting this interaction in a cellular context.

## Author Contributions

**Ibrahim Oluwatobi Kehinde**: conceptualization, methodology, formal analysis, investigation, visualization, writing – original draft. **Vuyisa Mzozoyana**: investigation, visualization, writing and editing, **Sizwe J. Zamisa**: investigation, visualization, writing and editing. **Mbuso Faya**: methodology, formal analysis. **Mahmoud E. Soliman**: conceptualization, review and editing, validation, supervision.

## Funding

The authors have nothing to report.

## Ethics Statement

This is a retrospective study that does not require approval from an ethics committee.

## Conflicts of Interest

The authors declare no conflicts of interest.

## Supporting information




**Supporting File 1**: cbdv70815‐sup‐0001‐SuppMat.docx

## Data Availability

The article does not include any research data, but all validations are thoroughly cited within the manuscript, and the results have been processed for analysis.
