# Supplementary material for: Targeting the TRIM28–EZH2 Protein–Protein Interface With Cysteine‐Reactive Covalent Inhibitors: A Computational Blueprint for Cancer Therapy
Source: Chem Biodivers. 2026 Jan 8;23(1):e02892. doi: 10.1002/cbdv.202502892 (PMC12781155; doi:10.1002/cbdv.202502892)
Supplement: Supplementary file 1 — Supporting File 1: cbdv70815‐sup‐0001‐SuppMat.docx [file CBDV-23-e02892-s001.docx]

**Protein-Protein Docking**

| **Cluster** | **Members** | **Representative** | **Weighted Score** |
| --- | --- | --- | --- |
| **0** | 174 | Center | -234.7 |
|  |  | Lowest Energy | -289.8 |
| **1** | 103 | Center | -229.3 |
|  |  | Lowest Energy | -285.7 |
| **2** | 64 | Center | -255.2 |
|  |  | Lowest Energy | -281.1 |
| **3** | 43 | Center | -247.2 |
|  |  | Lowest Energy | -266.6 |
| **4** | 38 | Center | -256.2 |
|  |  | Lowest Energy | -306.3 |
| **5** | 35 | Center | -233.8 |
|  |  | Lowest Energy | -264.0 |
| **6** | 35 | Center | -243.6 |
|  |  | Lowest Energy | -276.1 |
| **7** | 32 | Center | -244.2 |
|  |  | Lowest Energy | -276.7 |
| **8** | 29 | Center | -220.4 |
|  |  | Lowest Energy | -282.2 |
| **9** | 29 | Center | -234.0 |
|  |  | Lowest Energy | -265.8 |
| **10** | 27 | Center | -228.4 |
|  |  | Lowest Energy | -265.0 |
| **11** | 24 | Center | -234.8 |
|  |  | Lowest Energy | -255.9 |
| **12** | 21 | Center | -225.4 |
|  |  | Lowest Energy | -274.2 |
| **13** | 18 | Center | -228.6 |
|  |  | Lowest Energy | -307.6 |
| **14** | 18 | Center | -222.4 |
|  |  | Lowest Energy | -252.7 |
| **15** | 17 | Center | -220.7 |
|  |  | Lowest Energy | -263.0 |
| **16** | 17 | Center | -223.5 |
|  |  | Lowest Energy | -271.0 |
| **17** | 16 | Center | -231.7 |
|  |  | Lowest Energy | -254.9 |
| **18** | 15 | Center | -249.9 |
|  |  | Lowest Energy | -256.7 |
| **19** | 13 | Center | -220.9 |
|  |  | Lowest Energy | -239.8 |
| **20** | 13 | Center | -262.6 |
|  |  | Lowest Energy | -262.6 |
| **21** | 13 | Center | -252.5 |
|  |  | Lowest Energy | -252.5 |
| **22** | 12 | Center | -223.2 |
|  |  | Lowest Energy | -246.0 |
| **23** | 11 | Center | -227.3 |
|  |  | Lowest Energy | -286.7 |
| **24** | 10 | Center | -227.9 |
|  |  | Lowest Energy | -236.0 |
| **25** | 9 | Center | -221.4 |
|  |  | Lowest Energy | -249.0 |
| **26** | 9 | Center | -234.5 |
|  |  | Lowest Energy | -263.0 |
| **27** | 7 | Center | -223.3 |
|  |  | Lowest Energy | -241.1 |
| **28** | 5 | Center | -253.5 |
|  |  | Lowest Energy | -253.5 |
